# Development and evaluation of a strength-based method to promote employment of work-disability benefit recipients with multiple problems: a feasibility study

**DOI:** 10.1186/s12889-020-8157-3

**Published:** 2020-01-16

**Authors:** Kor A. Brongers, Bert Cornelius, Jac J. L. van der Klink, Sandra Brouwer

**Affiliations:** 1 University Medical Center Groningen, Department of Health Sciences, Community and Occupational Medicine, PO Box 196, 9700 AD Groningen, the Netherlands; 2 Center for Labour Expertise, Nijkerk, The Netherlands; 3 Dutch Social Security Institute: the Institute for Employee Benefits Schemes (UWV), Amsterdam, The Netherlands; 4 Research Centre for Insurance Medicine, AMC-UMCG-VU-UWV, University Medical Centre Amsterdam, Amsterdam, The Netherlands; 5 sTilburg University,Tilburg School of Social and Behavioural Sciences, Tranzo Scientific Center for Care and Walfare, Tilbrug, The Netherlands

**Keywords:** Disability benefits, Multiple problems, Return to work, Strength, Training program

## Abstract

**Background:**

For people with disabilities, chances to find or keep work are negatively affected by multiple problems like lower education, poverty and poor health. Furthermore, although active labour market policies proved to be effective for unemployed in general, success rates are poor for persons who are unemployed due to multiple problems. The present study aims to describe the development of a method as well as professional training to teach its application, and to assess the feasibility of method and training. The Strength-based method (CARm) aims to promote employment of work-disability benefit recipients with multiple problems.

**Methods:**

The main principles of the Strength model were redesigned for better applicability in a population of work-disability beneficiaries, resulting in the CARm method. As part of the CARm method, a training module for Labour Experts (LEs) was developed. To assess the new designed method and training, a one-group, pre-post design was used. Data were collected from eight participating LEs, five female and 3 male, aged between 41and 55 years and having 2–17 years working experience. We used self-report questionnaires and a semi-structured discussion meeting after the training sessions with the LEs.

**Results:**

Eight labour experts (LEs) from the Dutch Social Security Institute participated in the study. Most LEs felt an improvement in their ability to ascertain developmental needs, opportunities and threats in the client’s situation. Three months after the training, LEs almost unanimously agreed on the statements ‘I expect to use the CARm method more frequently in the future’ and ‘I use the CARm method in daily practice whenever possible’. The overall rating for the training on a scale from 1 to 10 was 7.6 (range 7–9). The overall satisfaction with the trainers was good.

**Conclusions:**

The CARm method and training was found to be a feasible approach to facilitate LEs working at the UWV reintegration service to support clients with multiple problems. Sufficient managerial support for participating LEs is a key factor for successful implementation of CARm. Results show that CARm is worth testing for efficacy in a future trial.

## Background

In many Western welfare states active labour market policies have been introduced, aimed at integrating the unemployed in general [1], and people with disabilities in particular, into the labour market [1, 2]. Although for unemployed benefit recipients active labour market policies proved to be effective [3, 4], for persons unemployed due to multiple problems these policies are much less successful [4].

For people with disabilities, chances to find or keep paid work are negatively affected by multiple problems like lower education, poverty and poor health [5]. Studies in the United Kingdom and the Netherlands show that individuals facing multiple problems, including poor health, have fewer chances to successfully return to work (RTW) than persons only facing unemployment [6, 7]. The review by Berthoud and colleagues (2003), focusing on how having multiple disadvantages affects employment, showed that nearly 10% of the half million included adults (aged 17 to 59) have multiple problems. These adults faced at least three out of six problems: no partner, low skills, impairment due to poor health, age over 50, high regional unemployment rate and ethnic minority. A model that examined the joint effect of multiple problems showed that persons with more problems ran a greater risk of being unemployed; for example, persons with six problems had a 90% risk of being unemployed [6]. In a Dutch study among persons needing social assistance, the proportion of those facing multiple problems was estimated to be 50–70% [7]. In line with the Dutch Central Bureau of Statistics, problems were differentiated into economic (i.e. no job, financial debts), cultural (i.e. low language skills, single parent, no starter qualification), normative (i.e. contact with police and justice, domestic violence, child abuse), and psychosocial (i.e. mental health problems, addiction, poor health) problems [8].

To address labour market inequalities and encourage employment of people with disabilities, in many OECD countries a number of employment support and rehabilitation programs are available. However, although some studies showed promising results, these interventions to help people on disability benefits to return to the labour market have little success [3]. To increase their effectiveness, these interventions need tailoring to the needs and skills of the people, and recognition of the existence of multiple disadvantages and how they affect employment chances [2, 7]. Furthermore, most interventions are problem-centred, i.e. focusing on problems, and seeking expert and compensatory support for each problem separately. Research on multi-problem families [9] and psychiatry [10] increasingly confirms that activation of people’s own strengths is an important tool for intervention, as they themselves may have personal and social resources, as well as strengths, to solve their problems.

In the Netherlands, the Comprehensive Approach to Rehabilitation (CARe) has been developed for use by mental health care professionals [11, 12], incorporating a strengths-focused approach. Based on equivalence it aims to improve the quality of life of persons with psychological or social vulnerabilities by focusing on their strengths, helping to realize their wishes and goals, and obtaining access to their living environment and social networks. Care is based on the Strength Model of Rapp, a well-known theoretical model from the 1980’s focusing on the personal qualities, talents, and strengths of persons with psychiatric disabilities, and on their environment [10]. The model includes six principles: (1) belief that these people can recover, reclaim and transform their lives; (2) focus on the individual’s strengths rather than deficits; (3) view of the community as an oasis of resources; (4) regarding the client as director of the helping process; (5) emphasis on the case manager/client relationship as primary and essential; (6) recognition of the community as the primary setting for our work. The Strength model has matured into a robust vision of mental health services, designed to facilitate a recovery-oriented partnership between client and practitioner. Although the model shows promising results its effectiveness is not undisputed. Ibrahim’s meta-analysis of clinical trials [13] did not report strong evidence for the effect of the strength-based model on level of functioning and quality of life. The authors were cautious in their conclusions, as is evident in their remarks: “the number of trials is low”, and “further evidence is required”. A more recent systematic review of research regarding the use of strength-based approaches in mental health service settings found emerging evidence that the utilisation of such an approach improves outcomes, including hospitalisation rates, employment/educational attainment, and intrapersonal outcomes such as self-efficacy and sense of hope [14]. Two studies measuring outcomes related to employment [15, 16] found that the practical and cognitive skills needed for social and occupational/vocational functioning significantly improved in the strengths group as compared to case management services routinely delivered by the mental health center [15]. Moreover, Stanard [16] found vocational/educational outcomes to be better in the experimental strengths group than in the control group.

Although developed for use in mental healthcare settings, the CARe method may also be suitable for vocational rehabilitation and disability settings, since it contains many elements (e.g. being strength-based, focused on clients’ wishes and goals, and involving activation of the environment) also likely to improve chances of re-employment of persons with multiple problems.

We therefore adapted the CARe method and developed the Comprehensive Approach to Reintegrate persons with Multiple Problems (CARm) for use by labour experts (LEs) at the Dutch Social Security Institute: the Institute for Employee Benefit Schemes (UWV). In the Dutch social security system, LEs play a key role in supporting the re-integration process of persons with a work disability and remaining workability. In general, the LE is responsible for the more complex clients with multiple problems. In current practice, in their role as work reintegration professionals, LEs focus mainly on the client and his or her limitations due to work disability. They have only limited time for contact with clients, and often only by mail or telephone. Yet people on work disability need opportunity to tell their story, and being heard may help them to reconnect with their environment [17]. The CARm methodology requires LEs to map the strengths of both the client and his/her environment, and to use these strengths to achieve the clients’ goals. CARm promotes personal contact, an integrated approach, and a focus on abilities rather than on pathology. To reinforce the efforts of LEs we added two modules, both aimed at strengthening the client’s motivation. As LEs are part of the social security system they may therefore not automatically be accepted by clients in their role as supporting professionals. Techniques focused on motivation can help to remove this resistance.

In this article we describe the development of both a method and professional training to teach application of the method, and to assess their feasibility. The aim of this assessment is to determine whether the CARm intervention is appropriate for further testing in a randomised controlled trial.

## Methods

The strength-based CARe method was adapted into CARm training (I), and its feasibility was investigated using Bowen’s framework (II).

### Development of CARm method and training

The objective of the developed method and training was to target partially disabled clients on work-disability benefit, facing multiple problems without an employer, and having remaining work capacity. The rationale of the CARm method is based on the main principles of the Strength model and consists of six steps: (1) building and maintaining a constructive helping relationship with the client; (2) collecting information and making a ‘strengths assessment’ with the client (this assessment can be used to gain an overview of a client’s former, current and desired situations in the fields of daily life, work, social contacts and leisure); (3) helping the client to formulate his/her wishes, make choices and set short- and long-term goals; (4) helping the client to acquire necessary resources to enhance his/her capabilities; (5) helping the client to execute the plan; (6) and (after completing the process) to learn, evaluate and adjust.

We organised three brainstorm sessions to define how these six principles could be included in the CARm method and what elements of the CARe method should be included. We first arranged a meeting with the authors of the method to explain and discuss our ideas and to obtain permission to adjust the method. Having received the authors’ approval we formulated a first concept of the CARm method. A second meeting was organised with five professionals with expertise in the development of reintegration programs and support of persons receiving unemployment- and work-disability benefits. In that meeting we discussed the concept of the CARm method and explained how we applied the Strength-based principles into CARm. The experts advised to focus on a specific group (i.e. recipients of work-disability benefits who had remaining work capacity), to involve LEs in the start-up phase as early adopters, and to start with a pilot study. The third meeting was organised with three UWV LEs who were eligible to be trained in the new method. These LEs were asked to reflect on the CARm method and its usability in daily practice. They endorsed the key elements of the Strength model and the CARm training itself, but they pointed out that some LEs have to adapt parts of their work routines and attitudes when using CARm in practice. Based on these meetings, the research team (KAB, BC, JJLvdK) developed a final version of the CARm method. To better apply the method in a population of work-disability beneficiaries we adjusted all terms and references related to psychiatry and psychiatric patients. An illustration taken from psychiatry was replaced by a reintegration case study from the daily practice of the first researcher (KAB), an LE as well as experienced reintegration professional. The original case study was an illustration of improved quality of life of a psychiatric patient, whereas the second case study is an illustration of the road to reintegration in work. This was more appropriate, as the overall goal of CARe is to improve patients’ quality of life, and the overall goal of CARm is clients’ reintegration into (paid) work. Finally, we added two modules on client-centred motivation and motivation against resistance.

#### CARm method

CARm is a method which enables LEs to systematically build an individual relationship with each client, aiming to support clients in their needs and to mobilise their social networks. The LE and client jointly develop a tailor-made plan for rehabilitation, aimed at work resumption. The LE drafts a *Personal Profile* of the client: information on the client’s current situation, needs, experiences, strengths, abilities and skills, and an inventory of external resources in the client’s social network. Based on this profile the client and LE then jointly develop a *Participation Plan* to set and prioritise goals, and to tackle the client’s problems.

#### CARm training module

As part of the CARm method a module was developed to train LEs of UWV. This training module focused on practical implementation of knowledge and skills. During a seven-day workshop, three whole days focused on theoretical knowledge regarding the CARm method, and four half days consisted of an active training module focused on the development of practical skills. The LEs received the book *Supporting recovery and* [18], and a training manual on the CARm method, written by the research team (KAB, BC, JJLvdK). To support LEs in their communication with, and especially motivation of, clients we added two modules, one dealing with tailor-made and client-centred motivation strategies, and the second dealing with motivation against resistance. The module on client-centred motivation strategies was inspired by the Situational Leadership Theory [19, 20]. This theory advocates that leaders adjust their leadership style to the levels of competence and commitment of their subordinates; leadership styles should not reflect the style of preference of the leader but the basic behaviour patterns seen in employees. Four leaderships styles are distinguished: Telling (with incompetent and uncommitted employees), Selling (incompetent but committed), Participating (competent but not committed) and Delegating (competent and committed). These leadership styles are comparable to the ‘frames of reference’ described by Eikenaar et al. [21], which aimed to describe the professional orientations of re-integration professionals in diverse settings. Dutch training situations have provided substantial experience in applying the Situational Leadership Theory to consultancy work and client counseling and coaching, therefore this application has been included as a module in the training for LEs.

The module on motivation against resistance was based on the general insight that resistance is a normal, human reaction when people are asked to change, especially when the new situation is perceived as a threat [22, 23]. Clients who are asked to change from benefit dependency to earning an income by working may feel insecure about their work capacity and their ability to earn an income. In this module LEs were trained to recognise resistance to change as an important factor behind stagnation, and to manage this accordingly. The first draft of the training manual was sent to the department of education of UWV. Two managers/trainers, not otherwise involved in this study, assessed whether the manual corresponded with UWV policy and the profession of the LE; they also assessed the educational quality of the training method. The training manual was subsequently presented to the authors of CARe for their comments, and final minor adjustments were made. The protocol of the training program is presented in Table 1.

**Table 1:** CARm training program: training activities and learning objectives

Day	Training activities	Learning objectives
1 (3 h) Practice	1. Trainer 1 introduces trainees to Strength-based method 2. Trainees list competencies they want to work on 3. Groups coached on how to draft *Personal profile* of clients under supervision of trainer 1	1: Trainees learn about Strength-based method 2: Trainees set goals to obtain required competencies 3: Trainees gain broader perspective on strengths and abilities of clients
2 (6 h) Theory	1: Trainees share success stories in working with clients. 2: Video shown to illustrate working based on strengths 3: Trainees interview client, under supervision of trainer 2 4. Trainees evaluate interview 5: Trainees discuss assignment: *Personal Profile*	1–3: Trainees experience focusing on clients’ skills, competencies and talents rather than deficits 4: Trainees learn from other trainees, trainer 2 and client how to incorporate Strength-based method in an interview 5: Trainees learn to better draft personal client profile
3 (3 h) Practice	1: Group coaching on individual questions from trainees. 2: Trainees present final *Personal Profile* and receive feedback from group and trainer 1	1: Trainees and trainer 1 reflect on competencies of trainees 2: Trainees learn to evaluate and improve final *Personal Profile*
4 (6 h) Theory	1: Trainer 2 introduces communication strategy (Hersey & Blanchard) (Newman) (Van der Klink & Terluin) 2: Trainees work in couples or in group on practical assignments on how to communicate adequately with clients 3: Trainers help trainees to work in supportive manner to construct holistic image and set goals with client 4: Assignment to work on *Participation Plan* with a client	1: Trainees obtain skills to improve communication with client 2: Trainees learn to motivate clients and build relationships with them 3: Trainees learn to focus on strengths rather than limitations or pathology 4: Trainees learn to collaborate with client on *Participation Plan* and to apply Strength-based method in practice
5 (3 h) Practice	1: Trainer 1 guides plenary discussion and responds to individual trainees’ questions about *Participation Plan* 2: Trainees present personal participation plans in the group, and receive feedback	1: Trainees obtain skills to improve *Participation Plan* 2: Trainees learn from experiences of other trainees on construction of *Participation Plan*
6 (6 h) Theory	1: Trainer 2 indicates importance of client’s natural environment 2: Trainees work with a scheme to map a client’s social network 3: Video illustrates a hostile and a supportive environment	1: Trainees know how to involve/activate social network of client 2: Trainees learn about importance of networks (family, professional, neighbourhood) 3: Trainees become aware of positive and negative influence of significant others
7 (3 h) Practice	1: Trainees present their process of cooperation with clients and reflect on goals formulated on first training day	1: Trainees learn from one another’s developments
Homework	Activities	Aim
	1: Trainees read literature provided for training day (Wilken & den Hollander, training manual) 2: Trainees draft personal profile and personal plan of randomly chosen client	1: Trainees obtain theoretical knowledge about rehabilitation and Strength model and start with equal level of knowledge 2: Trainees provide input related to daily practice

### Feasibility of CARm method and training

#### Assessing feasibility

To acquire more scientific knowledge on the applicability and effectiveness of CARm in disability settings, a feasibility study is an important first step. Feasibility studies are needed to determine whether an intervention is appropriate for further testing, to assess the potential success of implementation, and to uncover and reduce possible threats to validity [24]. The CARm method was assessed primarily in gatherings of experts, but also in meetings allowing for evaluation by LE’s who were attending the training.

To assess the feasibility of the CARm method and training we used a one-group, pre-post design. Data were collected with self-report questionnaires at baseline (T0; before the start of the training), directly after completion of each of the seven training days (T1-T7), directly after the end of the training (T8), and after three months (T9). A semi-structured discussion meeting with participating LEs was organised at T8 and chaired by the first author (KAB). We started the meeting with an open question to initiate the discussion, and then continued with more closed questions. At T9 a meeting of experts with the research team (KAB, BC) and the two trainers was organised to discuss any adjustments advised by the trainees.

We investigated the feasibility of the CARm method and training in line with the recommendations of Bowen et al. (2009). They identify the construct feasibility by means of a series of questions and methods [24]. For an intervention to be worthy of testing for efficacy, it must address the relevant questions within feasibility. It is also important to discard or modify those interventions that do not seem to be feasible according to data collected during the feasibility-study phase. Feasibility research in the intervention-research process is key to advancing only those interventions with a high probability of efficacy. Bowen recommends that investigators choose the area of focus that best matches the needs of the situation. In line with this recommendation we focused on aspects of feasibility which, in our view, best match the needs of the setting, community and population under study: acceptability, demand, implementation and practicality. Acceptability was operationalised as ‘the extent to which CARm is judged as satisfying to LEs and trainers, and the intent to continue use’; demand was operationalised as ‘the extent to which CARm is actually likely to be used by Les’; implementation was operationalised as ‘the extent to which CARm can be successfully delivered to intended recipients in a disability setting’; practicality was operationalised as ‘the extent to which LEs are capable of using CARm in daily practice’ and as ‘the extent to which LEs can implement the CARm in daily practice’.

#### Setting and participants

The feasibility study was conducted in collaboration with the regional UWV office servicing the northern region of the Netherlands. Data collection for this study started in April 2015 and follow-up was concluded in October 2015. Eligible for the present study were LEs of UWV working with unemployed clients on work-disability benefit and who, according to the UWV, have work capacity. All eligible LEs were informed by their district manager through a recruitment email. Since our aim was a feasibility study with maximum interaction and response, a maximum of eight LEs could participate in the CARm training programme [25]. The first eight volunteers were included. The actual training took place in the UWV office in Groningen, the Netherlands, from April to July 2015. Trainers were two certified experts from the RINO group (see Acknowledgements). Because of the scientific evaluation, participating LEs were asked to sign an informed consent form and all data were anonymised. The CARm training was accredited by the Dutch Association of Labour Experts. According to the Medical Ethics committee of the University Medical Center Groningen, ethical approval was not necessary for this study.

#### Measures survey

At T0 data were collected on background characteristics of LEs: age, gender, education, professional working experience and expectations.

To measure the quality of the training program we adapted a questionnaire developed by the University Medical Center Groningen, aimed at evaluating educational programs, to include Bowen’s four key aspects of feasibility: acceptability, demand, implementation and practicality. At baseline LEs were asked their opinion about 18 propositions regarding their current work methods and dealings with clients. At T1-T7 LEs were asked their opinion about the training content, expertise and teaching skills of the trainers. They were also asked to rate each training day on a scale of 0–10 and to propose any improvements. At T8 LEs were asked their opinion on content, design and organisation of the CARm training as a whole, to rate the whole training on a scale of 0–10 and to name strong points and points for improvement. At T8 and at T9 the LEs were asked whether the training and use of the CARm method had a lasting effect on their professional working methods. Propositions were recoded from a 5-point Likert scale scored 0 (disagree and totally disagree) or 1 (agree and totally agree) and missing (not applicable). An overview of the training is given in Table 1.

#### Measures semi-structured discussions

The semi-structured discussion meeting at T8 aimed to inventory trainees’ overall satisfaction with the method and training and whether the training should be adjusted. The following questions were discussed: *Were the periods between the training days sufficient for you to be able to work with your clients according to the CARm method? Has the CARm training sufficiently addressed the analysis and deployment of the social network of the client? Which key elements should be maintained and which elements should be omitted? What do you need from your employer UWV to be able to implement the CARm method in your daily practice?* In the experts’ meeting any adjustments advised by the trainees were discussed with the trainers. In both meetings notes were made by the researchers (KAB, BC) and the research assistant (JH).

### Statistical analysis

To describe the characteristics of participating LEs and the feasibility outcomes, we performed descriptive statistics, using SPSS version 20.0 (IBM Corp. Released 2011. IBM SPSS statistics Armonk, NY). Scores of opinions were dichotomised into ‘agree’ and ‘disagree’.

## Results

Eight LEs participated in this study. Their mean age was 47 years (range: 41–55, SD 5.6). Three LEs were male. Of the general population of LEs working for UWV, 34% are in the age category 45–54, 90% in the range of 35–64 years, and 47% are male. The baseline education of the LE is a bachelor’s or master’s degree followed by a one-year specialisation as LE. Of the eight participating LEs, seven had a bachelor’s degree and one had a master’s degree. Four LEs were educated in social work, two in economics, one in law and one in music. These education levels and different directions are in line with the whole population of LEs in the Netherlands. The average working experience as LE was 9.5 years (range: 2–17, SD 5.6). Four LEs were working in work- disability benefit claim assessment and four in reintegration service.

### Acceptability

Mean ratings of each training day ranged between 7.6 and 8.3. The mean overall rating for the entire training was 7.6 (range: 7–9). Of the 10 propositions regarding the quality of the training, presented immediately after the training, participants unanimously agreed on seven propositions, see Table 2.

**Table 2:** Acceptability of the CARm training for labour experts (*n* = 8) immediately after the training

Propositions	Agree (*n*)	Disagree (*n*)
The training fits well with my expectations	8	0
The training offers sufficient theoretical depth	8	0
The training offers sufficient opportunity to practice	7	1
The training offers sufficient opportunity for discussion	8	0
The discussion is informative.	8	0
I highly appreciate the training program	7	1
The prior information reflects the content well.	6	2
The training offers sufficient opportunity to ask questions	8	0
The training offers sufficient variety in teaching methods (e.g. lecture, interactive methods)	8	0
The training offers sufficient opportunity to learn different working methods	8	0

The overall satisfaction about the quality of the trainers was assessed with 9 propositions, presented at T2-T7. At T2-T6 the participants agreed unanimously on all propositions: ‘In general the presentation by the trainer is properly structured’, ‘The trainer formulates clearly and simply’, ‘The trainer gives sufficient insight into the problems of the study material’, ‘The trainer offers training material that suits the training goals well’, ‘The trainer is an expert on content’, ‘The trainer guides the group process well’, ‘The trainer explains clearly’, ‘The trainer is accessible’, ‘The trainer stimulates my learning process’ (not in table). At T7 one participant disagreed with one proposition: ‘The trainer offers training material that suits the training goals well’.

### Demand

Almost unanimous agreement on most propositions was observed. Immediately after the training LEs almost unanimously agreed on two propositions ‘As a result of the training I developed (or intent to develop) a different working method’ and ‘I will recommend the training to my colleagues’. Three months after the training (T9) LEs almost unanimously agreed on ‘I expect to use the CARm method more frequently in the future’, see Table 3.

**Table 3:** Demand and implementation of CARm for labour experts (n = 8) immediately after training and three months later

Propositions on demand	Agree (*n*)	Disagree (*n*)
*Immediately after the training*		
The training fits well with daily practice ^a^	6	1
During the training sufficient opportunity is offered for own input	8	0
The training offers sufficient opportunity to learn practical skills	7	1
As a result of the training I developed (or intent to develop) a different working method	6	2
I will recommend the training to my colleagues ^a^	5	2
*Three months after the training*		
I expect to use the CARm method more frequently in future	5	3
Propositions on implementation		
*Immediately after the training*		
I have the feeling that I control new skills	7	1
*Three months after the training*		
I use the CARm method in daily practice whenever possible	7	1
I find it difficult to make time to apply the CARm method in my daily work	4	4

^a^ 1 missing

### Implementation

Immediately after the training LEs almost unanimously agreed on ‘I have the feeling I control new skills’. Three months after the training LEs almost unanimously agreed on ‘I use the CARm method in daily practice whenever possible’. Four LEs agreed on ‘I find it difficult to make time to apply the CARm method in my daily work’; see Table 3.

### Practicality

LEs unanimously agreed on: ‘The practical assignment can be properly executed’ (practice days 1 and 3), ‘The practical assignment is a proper preparation for the study meeting’ (practice day 1), ‘The practice assignment properly integrates theory and practice’ (practice day 1), see Table 4.

**Table 4:** Practicality of CARm training and program for labour experts (*n* = 8) on practice and theory days per training day

Propositions	Practice day				Theory day		
	1	2	3	4	1	2	3
	Agree (*n)*	Agree (*n*)	Agree (*n*)	Agree (*n*)	Agree (*n*)	Agree (*n*)	Agree (*n*)
The practical assignment is clearly formulated	6	7^a^	6^a^	7	–	–	–
The practical assignment can be properly executed	8	7^a^	7^a^	8	–	–	–
The practical assignment is a proper preparation for the study meeting	8	7^a^	7^a^	6^a^	–	–	–
The practice assignment properly integrates theory and practice	8	7^a^	6^a^	7^a^	–	–	–
The training goals are clearly formulated	–	–	–	–	8	7	7^a^
The study material fits well with the training goals	–	–	–	–	8	8	6^a^
The study material fits well with the daily LE practice	–	–	–	–	7	8	6^a^
The provided literature fits well with the study meeting	–	–	–	–	8	8	6^a^

* 1 missing value

LEs unanimously agreed on ‘The training goals are clearly formulated’ (theory day 1), ‘The study material fits well with the training goals’ (theory days 1 and 2), ‘The study material fits well with daily LE practice’ (theory day 2), and ‘The provided literature fits well with the study meeting’ (theory days 1 and 2), see Table 4.

Three months after the training all LEs expected that the use of the CARm method would improve the professional quality of their work. Most LEs felt an improvement in their ability to ascertain developmental needs, opportunities and threats in the client’s situation. Furthermore, they felt better able to actively involve the client and his or her social network in the participation process, and to manage the process rather than the transfer of knowledge (not in table).

### Discussion meeting

During the semi-structured discussion meeting immediately after the training and the open questions: give 2 good points of the training and 2 points for improvement, the LEs expressed concerns about implementation. LEs believed the CARm method to be best suited for clients with complex problems and to require more time with a client than care as usual: time not only to attend the training and learn the method, but even more time with the client, to give them the opportunity to tell their story. Broad management support is therefore vital to implement the method. One of the LEs stated, “I wonder if I have enough time for this approach”. LEs also stated that social rehabilitation and work reintegration were not always clearly distinguished in the training. Quotes: “the emphasis on psychiatry is too strong”, “for me the aim is unclear; is it paid work or just participation?” and “I miss the link with work”. LEs advised making more use of learning materials in the training, such as videos focusing on work reintegration (“I miss the link with work”). Furthermore LEs stated that the CARm method fits better in the reintegration service of UWV (which allows multiple client contacts), than in the claim assessment service (which allows only one-time client contact). LEs stated that future CARm training should preferably involve LEs working in the UWV reintegration service.

## Discussion

### Main findings

This article describes the development of an innovative comprehensive approach for reintegration of persons on disability benefits and facing multiple problems (CARm), and its feasibility for intended use by LEs of UWV. As for the acceptability of the CARm training, the overall rating by participating LEs was 7,6 on a 1–10 response scale. With respect to training feasibility, the participants agreed unanimously on most propositions regarding the quality of the trainers. As for demand, most LEs stated that after the training they developed (or intended to develop) a different working method and expected to use the CARm method more frequently in the future. As for implementation (method feasibility), most LEs stated that they used the CARm method in daily practice whenever possible, although some found it difficult to make time to apply the method in their daily work. During the discussion meeting organised at the conclusion of the training, LEs further expressed concerns regarding implementation. They considered broad management support to be necessary for them to be able to apply CARm in daily practice and to make the method feasible. Further, regarding both theoretical and practical content, the training’s practicality was rated positively, its goals considered clear, and its study material found to fit well with the training goals.

### Strengths and limitations

To our knowledge our study is the first to study the feasibility of a strength-based and innovative integrated approach aimed at RTW of persons with multiple problems, such as unemployment combined with a work disability. Although most interventions are problem-centred, activation of people’s own strengths has been shown to be an important tool in intervention [9]. An important strength in the development of our study is its firm reliance on the internationally established Strength model of Rapp [26] and our collaboration with developers of a similar method and training, experts on reintegration instruments for unemployed persons on disability benefit, and practicing LEs of UWV. Another strength is the use of the framework of Bowen [24] to study the feasibility of CARm, as well as the use of a pre-post design.

As is inherent to any feasibility study, this study is limited in scale, scope and sample. Our results should therefore be interpreted with caution. With regard to generalizability,, there is a chance that the sample included more intrinsically motivated LEs since they participated voluntary, and could be characterised as innovators [27]. In addition, participating LEs were only recruited in offices of UWV servicing only the northern region of the Netherlands. This limited (non-representative) sample of LEs may have considered the CARm method and training to be more feasible than would non-participating LEs. Second, the questionnaire used to measure the quality of the training program and the feasibility has not been validated prior to the study, which may affect the quality of our findings.

### Comparison with other studies

This is the first study to investigate the feasibility of an integrated approach, based on the Strength model of Rapp, to be used in a social security setting and aimed at RTW of unemployed persons with disabilities. We therefore relate the results of this study to those conducted using a similar strength-based method in other populations, and to studies using another (but comparable) method in similar populations.

The feasibility of rehabilitation methods based on the Strength model is well established in mental health/psychiatry settings. This is illustrated by its association with positive results on different outcomes including decreased hospitalisation, improved quality of life, and improved social functioning [16, 28–30]. During the conduct of our study the results of another study, one on the effectiveness of CARe, a Strength-based method, were published [31]. Although this study reported an improved quality of life for clients, the difference between the intervention and control groups was not significant. Moreover, in our opinion the findings in this study are not generalisable to our study due to other sample characteristics, context and outcome measures. This study focused on a group of longstanding and severe impairments, especially severe mental illness (more than 72% of subjects were in sheltered living). Our sample included a less severely impaired and more heterogenic group of clients, most of whom were not in sheltered living. Where the CARe method (based on strength) has a strong and rather narrow clinical focus on mental health and improvement of quality of life, the adapted CARm method has a much broader biopsychosocial focus on participation in society (including work).

We promote more time for the client to tell his or her story in order to assess his/her need to participate in work. In line with the identity work process of van Hal et al. we believe that it is important that a client feels listened to and taken seriously.

A reintegration program more or less comparable to the CARm method is the participatory supportive RTW program [32]. This program is a complex intervention combining a participatory approach, in which unemployed persons on sick-leave develop an action plan for RTW with support of the LE of UWV, receive integrated care, and are placed in a competitive job. A process evaluation of that program for unemployed workers sick-listed due to musculoskeletal disorders showed good feasibility [32]. Execution of a comparable program for unemployed workers sick-listed due to a common mental disorder was less successful compared to similar programs evaluated in earlier studies [33].

### Implications for research and practice

Our study indicates that the CARm method might be an innovative comprehensive approach for LEs to support persons on disability benefits and facing multiple problems during their reintegration process, but strong management support is needed in advance. The study results will serve as a foundation for further research on the effectiveness of the CARm method, using a randomized-controlled-trial design (Dutch TRIAL register NL5626).

## Conclusion

The CARm method and training was found to be a feasible approach to facilitate LEs working in the UWV reintegration service to support clients with multiple problems. Sufficient managerial support for participating LEs is a key factor for successful implementation of this method, and thus for its validity. CARm is worthy of testing for efficacy in a future trial.

## Data Availability

Our Data Availability statement now reads: “The participant consent for the collection of data did not explicitly or implicitly include details of sharing their anonymised data. Due to the sensitivity of the data and the restrictions of informed consent, the data will not be stored in a public repository.” The data and meta-data will be stored in a repository at the UMCG, which ensures security and back-up of the data. The UMCG pursues a FAIR (Findable, Accessible, Interoperable and Re-usable) data policy for research conducted in the UCMG. We will include a description of the data in the data catalogue of the UMCG to make it available to others. Via the catalogue the data will be findable and available for researchers inside and outside the institute. A data access committee has been put in place, consisting of the principal investigators of the project, who will review requests to assure accessibility of the data. This access committee can be reached via the principal investigator, Prof. S. Brouwer; mail to: Sandra.brouwer@umcg.nl.

## Abbreviations

CARe: Comprehensive approach of rehabilitation
CARm: Comprehensive approach to reintegrate persons with multiple problems
LE: Labour expert
OECD: Organisation for economic co-operation and development
RTW: Return to work
UWV: Dutch social security institute: the institute for employee benefit schemes
